# Obituary

**Published:** 1854-02

**Authors:** 


*Obituary*—Died of typhoid fever at New California, Grant County, Wis., Josiah Stanly, M. D., on the 26th of Jan., 1854.

Dr. S. was a graduate of Rush Med. College, a young man of more than ordinary talent, and his prospects for future usefulness were to say the least flattering. His untimely death will be regretted by all who know him. He located in the above place after graduating last spring, where we learn he leaves a wife and two children with many friends to regret his early death.

J. F. H.

*Vaccine Virus.*

PHYSICIANS and others wishing to obtain a genuine article of Vaccine Virus, can do so, by remitting $1,00 by mail or otherwise to E. L. O'Hara, Druggist, No. 24 West Randolph St., Chicago.

